# Professional academic organizations on infectious disease Joint Statement on COVID-19

**DOI:** 10.4178/epih.e2020017

**Published:** 2020-04-29

Dear Fellow Citizens,

The world is experiencing great pain and confusion due to the COVID-19 that originated in China. The infection, which was limited to the Hubei Province in Wuhan, has spread to other parts of China. Now, patients have been confirmed on every continent, including 4 patients in Korea. As the number of patients in China is increasing rapidly, the number of inflow patients is expected to increase as well.

Concerned about the possibility of domestic transmission, the Korea Centers for Disease Control raised the level of response to the “orange” stage on January 27.

Infectious disease experts have been working hard in close cooperation with health authorities to anticipate the possibility of domestic influx and to prepare for and cope with an outbreak since it is known that a new type of pneumonia is occurring in Wuhan. Will Korea be safe in the future? How long will this epidemic last? Since the disease began in China, as of January 28, more than 5,500 people have been diagnosed. However, since the number of patients is continuing to increase and there are no effective treatments or preventive measures, it is hard to predict the magnitude of the influx of COVID-19 in neighboring countries, including Korea.

Depending on China’s control, it may take several months or more for this outbreak to end completely. If you believe you have symptoms, please be sure to contact your local public health center or the Korea Centers for Disease Control (phone 1339) for proper medical advice prior to your visit.

In Korea, from experience with SARS in 2003 and MERS in 2015, our professional academic organizations have experienced the importance of communication and cooperation between members and close collaboration with health authorities. In 2015, when the MERS crisis began there were no safe facilities and workforce to triage, diagnose, and treat patients. This was the first year the principles of infection safety were established and necessary facilities and resources were expanded. Based on the guidelines in each of the areas developed through trial and error, we will be able to wisely manage the current epidemic.

However, due to the lack of diagnostic tools and workforce supply in medical institutions and the novelty of COVID-19, the triage criteria for the patients are confusing and difficult. Overcoming this crisis with such limited resources is comparable to safely evacuating many people from a fire in a large building. To do this, health authorities, medical institutions, and the people must all help and follow each other. People coughing in public should observe good cough etiquette, such as wearing a mask, coughing on handkerchiefs or sleeves, and practicing good hand hygiene. Inadequate social fears caused by exaggerated or distorted information make it difficult for our community to cooperate and aim to expedite the response to the crisis. Based on scientific knowledge and experience gained from recent health crises, our professional academic organizations related to infectious diseases will cooperate with health authorities and work with people to put forth our best effort until this current crisis is overcome.

We ask for your continued interest and cooperation.

Thank you.

January 28, 2020

Korean Association of Infection Control Nurses, The Korean Society of Infectious Diseases, The Korean Society of Pediatric Infectious Diseases, The Korean Society for Preventive Medicine, Korean Society for Healthcare-associated Infection Control and Prevention, The Korean Society for Clinical Microbiology, Korean Society for Laboratory Medicine, Korean Society for Antimicrobial Therapy, Korean Society of Epidemiology

