# Supplementary material for: Professional academic organizations on infectious disease Joint Statement on COVID-19
Source: Epidemiol Health. 2020 Apr 29;42:e2020017. doi: 10.4178/epih.e2020017 (PMC7285421; doi:10.4178/epih.e2020017)
Supplement: Supplementary file 1 [file epih-42-e2020017-suppl.pdf]

Volume: 42, Article ID: e2020017

https://doi.org/10.4178/epih.e2020017

감염병 전문학술단체 신종코로나바이러스감염 담화문

국민 여러분,

세계는 지금 중국에서 시작된 신종 코로나바이러스 감염으로 인하여 큰 고통과 혼란을 경험하고 있습니다. 후베이성 우한시에 국한되었던 감염이 중국의 여러 지역으로 확산되면서 현재 세계 전 대륙에서 환자가 확진되었고 국내에서 환자 4명이 발생하였습니다. 현재 중국의 환자 발생이 빠르게 증가하고 있고 국내 유입 환자의 증가가 예측되며 확진자를 중심으로 국내 전파 가능성이 높아지는 것을 우려하여 1월 27일 질병관리본부는 "경계" 단계로 대응 수준을 격상하였습니다.

감염병 관련 전문가들은 우한에서 원인미상의 폐렴이 발생하고 있다는 것이 알려진 시점부터 국내 유입 가능성을 예측하여 이에 대한 대비ㆍ대응을 위하여 보건당국과 긴밀한 협조 하에 최선의 노력을 해오고 있습니다. 앞으로 과연 우리나라는 안전할까요? 그리고 언제까지 이 유행이 지속될까요? 질병이 시작된 중국은 1월 28일 기준 전 지역에서 5,500여 명이 진단되었으나 효과적인 치료제나 예방수단이 없는 상황에서 환자가 계속 발생하고 있기 때문에 우리나라를 포함한 이웃나라의 유입 감염의 규모는 어떠할 것인지 감히 예측하기 어렵습니다. 중국의 통제 상태에 따라서 완전 종식까지는 수개월 이상 소요될 가능성도 있습니다. 만약 스스로 증상을 인지하는 경우 의료기관 방문 전 반드시 관할 보건소나 질병관리본부(1339)로 연락하셔서 적절한 진료 안내를 받으시도록 유념해주시기 부탁드립니다.

국내에서는 이미 2003년 사스와 2015년 메르스의 경험을 통해서 저희 전문학술단체들은 회원들간의 소통 협력과 보건당국과의 긴밀한 협업이 매우 중요함을 경험하였습니다. 특히 환자를 분류

Volume: 42, Article ID: e2020017

https://doi.org/10.4178/epih.e2020017

하여 진단하고 치료할 수 있는 안전한 시설과 인력이 전혀 없는 상태에서 메르스 사태가 시작되었던 2015년은 오히려 이후 감염 안전에 대한 원칙을 다지고 필요한 시설과 자원을 확충하는 원년이 되었고 여러 가지 시행착오를 경험하며 얻게 된 각 분야의 지침을 바탕으로 우리는 지금의 신종감염병 재난을 슬기롭게 극복할 수 있을 것입니다.

그러나 지금은 의료기관의 진단수단, 인력 공급이 부족하고, 새로운 감염병이기 때문에 환자를 분류하는 기준도 혼란스럽고 어렵습니다. 이토록 제한된 자원으로 이 위기를 극복하는 것은 마치 화재가 난 큰 건물에서 모든 사람들이 한 명도 빠짐없이 무사히 탈출하는 것과도 같습니다. 그러기 위해서는 보건당국과 의료기관과 국민들이 모두 서로 돕고 잘 따라 주셔야 합니다. 공공장소에서 기침하는 사람은 마스크를 쓰는 것, 기침을 할 때 손수건이나 소매에 대고 기침하는 것, 손위생을 잘하는 것 등으로 표현되는 기침 예절을 잘 지켜야 합니다. 과장되거나 왜곡된 정보로 인하여 부적절하게 초래되는 사회적 공포는 방역당국의 신속한 대응과 위기 극복을 위한 우리 공동체의 협력과 노력을 힘들게 만듭니다. 저희 감염병 관련 전문학술단체들은 최신지견의 과학적인 지식과 경험을 토대로 위기를 극복할 때까지 보건당국과 협력하여 최선의 노력으로 국민과 함께 할 것입니다.

국민 여러분의 많은 관심과 협조 부탁드립니다.

감사합니다.

2020년 1월 28일

대한감염관리간호사회 대한감염학회 대한소아감염학회 대한예방의학회 대한의료관련감염관리학회 대한임상미생물학회 대한진단검사의학회 대한항균요법학회 한국역학회 (가나다 순)
